# Adaptation to macrophage killing by *Talaromyces marneffei*

**DOI:** 10.4155/fsoa-2017-0032

**Published:** 2017-06-30

**Authors:** Monsicha Pongpom, Pramote Vanittanakom, Panjaphorn Nimmanee, Chester R Cooper, Nongnuch Vanittanakom

**Affiliations:** 1 Department of Microbiology, Faculty of Medicine, Chiang Mai University, Chiang Mai, Thailand; 2 Faculty of Medicine, University of Phayao, Phayao, Thailand; 3 Faculty of Medical Technology, Huachiew Chalermprakiet University, Samut Prakan, Thailand; 4 Department of Biological Sciences, Youngstown State University, Youngstown, OH, USA

**Keywords:** heat stress, intracellular survival, macrophages, nitrosative stress, nutrient starvation, oxidative stress, *Talaromyces marneffei*

## Abstract

*Talaromyces* (*Penicillium*) *marneffei* is an important opportunistic fungal pathogen. It causes disseminated infection in immunocompromised patients especially in Southeast Asian countries. The pathogenicity of *T. marneffei* depends on the ability of the fungus to survive the killing process and replicate inside the macrophage. Major stresses inside the phagosome of macrophages are heat, oxidative substances and nutrient deprivation. The coping strategies of this pathogen with these stresses are under investigation. This paper summarizes factors relating to the stress responses that contribute to the intracellular survival of *T. marneffei*. These include molecules in the MAP signal transduction cascade, heat shock proteins, antioxidant enzymes and enzymes responsible in nutrient retrieval. There is speculation that the ability of *T. marneffei* to withstand these defenses plays an important role in its pathogenicity.

## Lay abstract

*Talaromyces marneffei* is an important dimorphic fungus that causes disease in immunocompromised patients. The pathogenicity of *T. marneffei* depends on the ability of the fungus to survive the killing process and replicate inside the host macrophage cells. This paper summarizes factors relating to the stress responses that contribute to the intracellular survival of *T. marneffei*. There is speculation that the ability of *T. marneffei* to withstand these defenses plays an important role in its pathogenicity.

*Talaromyces marneffei* (formerly named *Penicillium marneffei*) is a dimorphic fungus which is endemic in Southeast Asian countries and southern China. This fungus is able to grow either in a filamentous form at 25–30°C or a yeast-like form at 37°C [1,2]. *Talaromyces marneffei* grows as yeast form inside the host body [3]. It causes an infection in both immunocompetent and immunocompromised patients [4]; however, most cases of infection occur in immunocompromised hosts [5]. The first review of case reports regarding *T. marneffei* infections was a series of patients from Thailand during the early AIDS era [6]. Since then, the incidence of infection has increased concomitantly with the AIDS pandemic, especially in Southeast Asia [7–15]. Among these various cases, the infection has also been detected in immunocompromised people who visited the endemic countries. Currently, however, the rate of infection has decreased dramatically due to antiretroviral applications in HIV-infected patients, but the infection groups have shifted to include non-HIV immunocopromised patients [16], suggesting the importance of the need for global health awareness of *T. marneffei* infection.

Typically, the symptoms of disseminated infection due to *T. marneffei* are fever, weight loss and multiple organ dysfunction including pancytopenia, hepatosplenomegaly and respiratory symptoms. Cutaneous manifestation is sometimes presented [17–23]. There are differences between *T. marneffei* infection in AIDS patients and other acquired immunodeficiency syndromes. These differences include variation in the type of organ affected, clinical presentation and disease progression [24]. In HIV-infected patients, *T. marneffei* infection usually causes high persistent fever, dyspnea, hepatosplenomegaly and skin lesions. Occasionally, infection of the CNS is also observed [25]. The clinical presentations in cases of infection in non-HIV infected patients are generally similar but show a low degree of multiple organ dysfunction. Generalized lymphadenopathy presented in this group, in conjunction with increases in neutrophil counts and CD4/CD8 ration, implies that there are specific immune responses to *T. marneffei*. Aberrant symptoms such as pleuritis and osteomyelitis were also found in some non-HIV infection patients.

*Talaromyces marneffei* is a facultative intracellular pathogen, and it grows as a fission yeast inside the macrophages while appearing extracellularly as elongated arthroconidia-like yeast cells. Postulation on the sequence of events for establishing the infection has been made from ultrastructural observation from the histopathology of *T. marneffei* infected patients. Importantly, the fate of the conidia after being engulfed by the host macrophage has been documented both in immunocompromised and immunocompetent hosts [3]. It has been confirmed that the fungus can survive and replicate inside the phagolysosome, then subsequently escape from the phagosome into the cytoplasm. This evidence revealed that *T. marneffei* has the stress tolerance ability to resist the phagosomal killing mechanisms. However, the detailed knowledge of this resistance mechanism has not been well described in this particular fungus.

The first important mechanism which enables the fungus to establish infection is the conversion of conidia to the yeast phase. This concept is supported in reviews that concluded that the deletion of genes involved in the phase transition altered the host response both *in vitro* and in a macrophage infection model [26,27]. Examples of such genes include the following: *abaA*, which is involved in asexual development and yeast growth [28]; the genes encoding for Ras and Rho GTPases, which are involved in the yeast and hyphal morphogenesis [29]; *pakA*, which controls conidial germination and polarized growth of the yeast-like cells [30]; and *pakB*, which is required for an inhibition of yeast-like cell morphogenesis at 25°C [31].

A second mechanism deserving significant consideration is its resistance to phagocytic killing. The intracellular survival inside the macrophage of this fungus had been shown both in natural infection [3] and in *in vitro* observations [32,33]. Electron micrographs showed both dead conidia and multiplied yeast cells inside the macrophages. Inhibition of phagosome maturation contributing to the intracellular survival has been demonstrated in the RAW267 murine macrophage infection model [33]. Additionally, some of the genes relating to oxidative and heat stress responses have been reported, for example, genes coding for catalase-peroxidase (CpeA) [34] and Hsp30 [35]. Their transcripts were found to accumulate in the conidia and are upregulated in the yeast form, suggesting the potential role of these proteins in the yeast pathogenic phase of this fungus. In this review, we summarize several factors that contribute to the intracellular survival of *T. marneffei*. However, in-depth information on fungal factors that contribute to resistance to the phagocytic killing in *T. marneffei* and associated molecular mechanisms are still lacking, thereby warranting more attention.

## Resistance to oxidative stress by *T. marneffei*

The main antimicrobial mechanisms of the macrophages are the generation of oxidants and acidification of the phagosomes [36]. The macrophages destroy fungal pathogens through the creation of a harsh environment where normal metabolism is difficult and also through the activation of hydrolytic enzymes which degrade the pathogen. To achieve acidification, the vacuolar H^+^-ATPase at the phagosomal membrane facilitates the pumping of H^+^ into the phagosome. The phagosomal H^+^ further combines with superoxide (O_2_^-^) produced by the activity of NADPH oxidase. Several reactive oxygen species (ROS) that are toxic to the *T. marneffei* cells are then generated such as hydrogen peroxide (H_2_O_2_) and hydroxyl radicals (OH^-^). Nitric oxide synthase facilitates the production of nitric oxide (NO) in the phagosome. Upon reaction with oxygen radicals produced by the NADPH oxidase, NO is converted to reactive nitrogen species (RNS), which are extremely toxic. Together, these ROS and RNS can damage the DNA, lipids and proteins of the pathogen inside the phagosome. The macrophage can produce almost 60 μM and up to 14 mM H_2_O_2_. These are fungicidal concentrations for most nonpathogenic fungi [37].

The importance of NO in the killing of *T. marneffei* has been documented in mouse macrophages [38]. NO has been reported as being more efficient than the superoxide anion, one of the ROS, in giving protection against *T. marneffei* [39]. Additionally, activated murine macrophages failed to eradicate the engulfed conidia after treatment with N-monomethyl-L-arginine which inhibits NO synthesis, confirming the role of NO in the killing mechanism [40]. *Talaromyces marneffei* has also been shown to be susceptible to the respiratory burst toxic products in phagocytic killing assays [41–44]. These data from these investigations demonstrate that both ROS and RNS are essential for host resistance to infection from *T. marneffei*. However, the direct role of NO against *T. marneffei* infection has not been documented.

Indeed, *T. marneffei* has a relatively low resistance to H_2_O_2_ when compared with other intracellular fungal pathogens. This was shown when *T. marneffei* cells were spotted on a medium containing H_2_O_2_ and the fungus allowed to grow at either 25°C or 37°C for 5–7 days. Survival occurred only on media containing less than 3 mM of H_2_O_2_ [45]. In contrast, other pathogenic fungi demonstrated very high resistance to this chemical. For example, *Candida albicans* exhibited natural resistance to 10–50 mM H_2_O_2_ [46,47]. The yeast phase of *Paracoccidioides brasiliensis* also survives in very high concentrations of H_2_O_2_, up to 100 mM [48]. The intriguing question is how can *T. marneffei* survive and replicate inside the phagosome of the macrophage in immunocompetent hosts despite having a low resistance to H_2_O_2_. It is highly possible that phagocytes in the immunocompromised hosts might produce insufficient quantities of antimicrobial substances. However, there is no evidence to support what concentration of the oxidative substances would be produced by the macrophages of immunocompromised compared with immunocompetent patients. Infection by *T. marneffei* conidia in mouse peritoneal macrophages revealed that there was no difference in phagocytosis, killing or phagosome maturation between the peritoneal macrophages in normal mice and cyclophosphamide-induced immunocompromised mice [33]. In this same study, the experimental data also showed that the fungus could inhibit phagosome–lysosome fusion. Therefore, it is very interesting to clarify the inhibition mechanism of phagosome–lysosome maturation in the *T. marneffei* infected human macrophage model. Additionally, *T. marneffei* could secrete antioxidant molecules to neutralize ROS/RNS toxicity. A significant upregulation of a transcript encoding for catalase-peroxidase (CpeA) bifunctional enzyme by the yeast form of *T. marneffei* is shown in the yeast culture and during infection in murine macrophages [34,45]. Thus, this enzyme may play an important role by limiting the effect of H_2_O_2_ during infection.

Ultrastructural observation demonstrated the presence of the fission yeasts of *T. marneffei* inside the phagosomal compartment of the macrophages, implying that the fungus fully adapts to survive and replicate before escaping from the harsh environment of phagosome into the less toxic cytoplasmic environment [3]. In the phagosome, the fungus must deal with heat, oxidative and nutrient deprivation stresses. The molecular mechanisms for stress response in *T. marneffei* that have been studied to date are described below.

## The regulation of fungal response to oxidative stress

There are two control mechanisms that fungi generally use to challenge oxidative stress; nuclear localization of transcription factors and signal transduction via phosphorylation [49]. In the case of nuclear localization, stress can cause transcription factors to translocate from cytoplasm to the nucleus, subsequently resulting in increased expression of genes involved in the oxidative stress response. One type of fungal transcription factor that plays a role in oxidative challenge is Yap1 and its orthologs. Yap1 is a basic region leucine zipper containing protein of *Saccharomyces cerevisiae* [49]. Under nonstress conditions, cytoplasmic Yap1 interacts with importin proteins (Imp) via its nuclear localization signal at an amino terminal and is delivered to the nucleus. Inside the nucleus, Yap1 dissociates from Imp and binds to an exportin protein (Crm1) and GTP-loaded Gsp1 via a nuclear export signal at the carboxyl terminal and is exported to the cytoplasm. Under conditions of oxidative stress, the carboxy-terminal cysteine-rich domain of the Yap1 molecule is modified. This modification leads to the sequestration of nuclear export signal and allows Yap1 to accumulate inside the nucleus. Nuclear accumulation of Yap1 results in the increase of target gene expression such as thioredoxin (trx2), γ-glutamylcysteine synthetase *(gsh1)* and glutathione reductase *(glr1)* that are crucial for H_2_O_2_ tolerance [49,50]. In the pathogenic fungus *C. albicans*, deletion of *cap1*, a *yap1* homolog, results in hypersensitivity to oxidative stress induced by diamide and H_2_O_2_ [51]. In addition, *C. albicans cap1* transcription is induced in the presence of human neutrophils suggesting that it has a role in the response of the fungus to host immune cells [51]. The *yapA* (*yap1* homolog) has recently been characterized in *T. marneffei*. Deletion of the *yapA* was hypersensitive to oxidative and nitrosative stress. This mutant exhibited decreased survival inside a human macrophage cell line compared with the wild-type and the complemented strain [52]. Hence, *yapA* appears to have a role in the stress response of *T. marneffei*.

**Figure 1.**
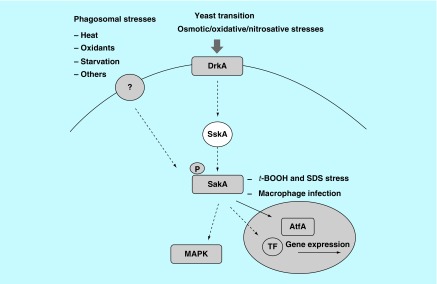
**Phosphorylation pathway in response to stresses in *Talaromyces marneffei*.** The DrkA and other histidine sensor kinases respond to the environment signals including stress from osmotic, oxidation and heat. The signal is transferred to the MAP kinase, SakA. After phosphorylation, SakA translocates into the nucleus where it interacts with the downstream effector AtfA or other unidentified transcription factors which activate the transcription of stress response genes. In addition to nuclear translocation, phosphorylated SakA may activate other molecules in the MAPK cascade in response to the stimuli.

With regard to stress-response mechanisms involving signal transduction systems, one of the conserved pathways that are used in eukaryotes to respond to the environmental stimuli includes the MAPK cascades [53]. MAPK pathways regulate the response to a variety of cellular activities including proliferation, differentiation, cell death and homeostasis. In the yeast *S. cerevisiae*, there are five MAPK cascades that regulate gene expression in response to mating, filamentous growth, high osmolarity response, maintenance of cellular integrity and ascospore formation [54]. The highly conserved structure of MAPK pathways is composed of three kinases that transfer the signals by phosphorylation. Through cellular signals, the MAP kinase kinase kinase (MAPKKK) is phosphorylated and stimulates the phosphorylation of the MAP kinase kinase (MAPKK). Phosphorylated MAPKK causes the phosphorylation of the MAP kinase (MAPK) on both a threonine and a tyrosine conserved residue [55]. After phosphorylation, MAPK is able to phosphorylate its substrates including transcription factors and cellular proteins in response to the stimuli [53]. MAPK pathways that play a role in response to diverse forms of stress such as oxidative stress, heat shock, osmotic and nutrient starvation, are termed as stress-activated protein kinases (SAPKs). SAPK cascades are identified in several yeasts and pathogenic fungi including the Hog1 pathway in *S. cerevisiae*, the Spc1/Sty1 pathway in *Schizosaccharomyces pombe* and the CaHog1 pathway in *C. albicans* [53]. The Spc1/Sty1 pathway in *S. pombe* is associated in core environmental stress responses to oxidation, osmotic effects, heat shock stress, UV irradiation, alkylating agents and heavy metal toxicity [56]. The system is composed of Wis4 or Win1 (MEKK), Wis1 (MEK) and the MAP kinase Sty1. Under conditions of oxidative stress, Sty1 is phosphorylated at threonine and tyrosine residues within the activation loop. Phosphorylated Sty1 in the cytoplasm is translocated to the nucleus and subsequently phosphorylates and stimulates a basic region leucine zipper transcription factor, Atf1 [53,56]. Atf1 activates the expression of genes encoding for cytosolic catalase (*ctt1*), glutathione peroxidase (*gpx1*), thioredoxin reductase (*trr1*) and glutathione S-transferase (*gst1*, *gst2*, *gst3*). These genes function as part of the fungal adaptation to oxidative stress. In the filamentous fungus *Aspergillus nidulans*, the *sakA* gene is a *sty1* homolog involved in oxidative and heat stress signal transduction [57]. In addition, the interaction of SakA and AtfA (Atf1 homolog) regulates *A. nidulans* gene expression during stress and conidiophore development [58]. In *T. marneffei*, the *sakA* and *atfA* genes have been studied [59–61]. The *sakA* gene is associated with asexual development and yeast cell formation *in vitro* and is necessary for the viability of *T. marneffei* conidia under heat, oxidative and nitrosative stresses [59,61]. Additionally, the *sakA* is also involved in a wide variety of cell activities including yeast morphogenesis, sporulation and red pigment formation [61]. In addition, both *sakA* and *atfA* gene deletion resulted in the decreased survival of conidia inside human macrophage cell line, suggesting they may have a role in protecting the conidia from macrophage killing [59,60]. The interaction of SakA and AtfA under conditions of stress has been proposed to occur in *T. marneffei*. A recent study revealed that SskA (the Ssk1p homolog in *S. cerevisiae*) response regulator is required for the phosphorylation of SakA [62]. A summary of the SAPK signaling cascade in *T. marneffei* that has been identified to date is shown in Figure 1.

The system that transmits the environmental and cellular signals to SAPK cascades is called the two-component signaling system. In prokaryotes, this system is composed of a sensor kinase and a response regulator [53]. In eukaryotes, the sensor histidine kinase which is often a transmembrane protein contains both a histidine kinase domain and a receiver domain within the single polypeptide chain [63]. The phosphate group is first transferred from a histidine residue in the kinase domain to an aspartate residue in the receiver domain. The phosphate is subsequently transferred to a histidine residue present on a histidine-containing phosphotransfer (Hpt) protein and to an aspartate residue in the molecule of a second response regulator [63]. The best characterized phosphorelay system in eukaryotes is the Sln1 pathway that transmits the signal to the Hog1 cascade [63]. This pathway is comprised of the Sln1 sensor kinase, the Ypd1 phosphotransfer protein and Ssk1 response regulator. Under stress conditions, the Sln1–Ypd1–Ssk1 phosphorelay system transmits the signals to the Hog1 pathway. In *S. cerevisiae*, only one histidine kinase has been identified, whereas several had been isolated in other fungi: *C. albicans* and *S. pombe* have at least three, *Cryptococcus neoformans* has seven and *A. nidulans* has 15 [64]. Among the dimorphic fungi, *Blastomyces dermatitidis* and *Histoplasma capsulatum*, a Drk1 histidine kinase distinct from the Sln1 in *S. cerevisiae* has been identified. The *B. dermatitidis* and *H. capsulatum drk1* mutants are unable to convert to yeast growth at 37°C and exhibit decreased pathogenicity in a mouse model of pulmonary infection [65]. In *T. marneffei*, homologs of both Sln1 and Drk1 have been identified and denoted SlnA and DrkA, respectively. SlnA and DrkA are required for osmotic stress adaptation and are involved in the phosphorylation of MAP kinases, SakA and MpkA, during osmotic and cell-wall stress. In addition, SlnA and DrkA are also associated with conidial germination and dimorphic switching during macrophage infection, respectively [66].

Another protein that can receive the phosphate group from the Ypd1 phosphotransfer protein is an Skn7 response regulator. Unlike Ssk1, Skn7 contains a DNA-binding domain and functions as a transcription factor. After phosphorylation by Ypd1, Skn7 is translocated to the nucleus and regulates the expression of antioxidant genes independently of the Hog1 pathway [51,53]. The role of Skn7 on oxidative stress has been studied in *C. albicans*, *C. neoformans*, *A. nidulans* and *Aspergillus fumigatus* [67]. This protein is also associated with morphogenesis in *C. albicans* and *A. nidulans* and is required for melanin production, sexual reproduction and virulence in *C. neoformans*. In addition, the *T. marneffei skn7* gene is able to functionally replace Skn7 in *S. cerevisiae* suggesting it has a role in the oxidative stress response in this dimorphic fungus [68].

## Proteins associated with the phagosome-mimic stress response

Several stress response proteins of *T. marneffei* have been reported. Their functions are discussed below with regard to those stress conditions that mimic those found within phagocytic cells.

### Antioxidant proteins

Phagosomal oxidative stress generally relates to the actions of ROS and RNS. *T. marneffei* exhibits a moderate resistance to H_2_O_2_. *In vitro* experiments showed that this fungus is killed by H_2_O_2_ at concentrations of 3–5 mM. This amount of H_2_O_2_ is relatively low compared with the lethal concentration for other pathogenic fungi. The low level of tolerance in *T. marneffei* is comparable to the dose reported in the nonpathogen *S. cerevisiae*. Therefore, the ability of *T. marneffei* to survive inside the phagosomal environment should be due to the combination of several unknown factors rather than oxidant-resistance capability alone, for instance, the capability of the fungus to inhibit phagosome and lysosome fusion. For this reason, there is a need for more extensive studies on the mechanism(s) that facilitate the intracellular survival of this fungus.

Discovery of several detoxifying enzymes implies that *T. marneffei* possesses an efficient and adequate system to cope with the phagosomal environment of the macrophage. Two genes encoding for antioxidant proteins have been reported. They include superoxide dismutase (*sodA*) [69] and catalase-peroxidase (*cpeA*) [34,45]. These antioxidant proteins have been shown to aid resistance to phagocytic killing by fungal pathogens such as *H. capsulatum* and *C. neoformans* [70,71]. In *T. marneffei*, only the involvement of *cpeA* in pathogenicity has been examined. When a *cpeA* deletion mutant and the wild-type strain were used to infect a THP-1 macrophage cell line, the mutant exhibited a decreased ability to survive. This result suggested that the CpeA plays a role in the resistance to phagocytic killing [45].

Another important antioxidative molecule is melanin. Melanin has been reported as having protective functions to oxidative and other types of cell-wall stresses in the fungal pathogens [72]. The *T. marneffei* genome contains genes encoding polyketide synthases responsible for DHN-melanin synthesis [73]. Melanization of *T. marneffei* has been demonstrated to assist oxidative and heat resistance *in vitro* [74,75].

It is important to note that the general activities of the antioxidant factors described above were observed mainly *in vitro*. These studies still lack proof through the use of animal models *in vivo*. In addition, there are several enzymes that have not been characterized with regard to their expressions during the yeast phase of *T. marneffei*, but the genes for these potential antioxidant proteins have been found in the genome of this organism.

### Heat responsive proteins

One of the classic virulence factors of pathogens is the ability to tolerate human body temperature. *Talaromyces marneffei* is a dimorphic fungus which responds to a human body temperature by initiating yeast morphogenesis, which is tightly linked to the virulence of the organism. As it is already known in eukaryotes, heat shock proteins promote folding of aggregated or denatured proteins in order to adapt to elevated temperatures [76–78]. Heat shock proteins (Hsp) play an important role in the adaptation process of pathogenic fungi during infection [79]. An important example had been described in the study of the role of small heat shock proteins Hsp12, Hsp20 and Hsp30 in the pathogenesis of the plant pathogen *Ustilago maydis* [80]. In the human pathogen *C. albicans*, Hsp21 is required for resistance to neutrophilic killing through the regulation of glycogen production and trehalose homeostasis in response to elevated temperatures [81]. Hsp70 modulates the interaction of *C. neoformans* with human alveolar epithelial cells and decreases macrophage killing [82]. It has been shown that *T. marneffei* produces a large amount of antigenic Hsp30 and Hsp70 after conidia encounter a temperature upshift to 37°C [35,83]. Hsp30 is of particular interest since it is accumulated at high levels in conidia, suggesting its importance during germination. The latter is believed to be the first stage of infection after the propagules reach the human alveoli. Proof of this hypothesis awaits investigation and subsequent studies with an *hsp30* deletion mutant. Given that Hsp60 and Hsp90 are also preferentially expressed in the yeast phase of *T. marneffei* [84], similar studies with mutants in these proteins would be of interest.

Other members of the heat shock protein family are also present in the genome of *T. marneffei* [genome analysis, unpublished data]. Given the temperature-dependent dimorphic nature of *T. marneffei*, these heat shock proteins are likely to play important roles in the pathogenesis of this dimorphic fungus. The functional characterization of these proteins is also warranted.

### Nutrient starvation responsible proteins

As glucose is either limited or absent in the phagosomal niches, *T. marneffei* cells must adapt to the stress of glucose starvation, but at the same time attempting to exploit alternative carbon sources within the host-cell environment. Glucose is normally present at low concentrations in the phagosome while this niche contains complex mixtures of alternative carbon sources from host such as amino acids, carboxylic acids, such as lactate and fatty acids [85]. For intracellular survival, *T. marneffei* has to assimilate these alternative carbon sources for energy production.

The glyoxylate cycle has been shown to be induced during *T. marneffei* infection, thereby contributing to the virulence of this fungus [86]. This pathway uses acetyl-coA from the tricarboxylate cycle as an energy source. The gene *acuD*, encoding for isocitrate lyase, is a key enzyme in the glyoxylate shunt pathway. Expression of this enzyme is increased in the yeast phase of *T. marneffei* and during macrophage infection [87]. Also, genes encoding for important gluconeogenesis enzymes, including fructose-1,6-bisphosphatase, have been isolated from a yeast phase cDNA library. These genes are highly expressed during yeast morphogenesis [88].

In addition, the amino acid tyrosine facilitates the acquisition of carbon via the gluconeogenesis pathway [89]. Tyrosine can be changed into fumarate and acetoacetate and then catabolized to acetyl-coA, a substrate of the tricarboxylic cycle, for energy production. In addition to the nutrient supply, tyrosine catabolized products are used for pyomelanin production. As mentioned earlier, *T. marneffei* produces melanin molecules that play the role in pathogenicity [74,75].

In response to increasing the activity of gluconeogenesis, *T. marneffei* decreases glycolytic activities which use glucose as a substrate. Expression of a glycolytic enzyme, glyceraldehyde-3-phosphate dehydrogenase (*gpdA*), decreased during macrophage infection [90]. Together, the data from these findings suggested that *T. marneffei* responds to glucose starvation and compensates for energy production by reduction in glycolysis and induction of the glyoxylate shunt pathway and gluconeogenesis.

Finally, *T. marneffei* must encounter the absence of suitable sources of nitrogen and micronutrients, including iron, within the host cell. Iron has been shown to be important to the growth and pathogenicity of this fungus. Iron overload enhances the intracellular and extracellular growth of *T. marneffei* [90]. Additionally, the depletion of intracellular iron within the phagosome, by using the antimalarial drug chloroquine, inhibited the growth of this fungus [91]. However, the detailed mechanism(s) of iron acquisition by *T. marneffei*, which also compete with the presence of host iron-binding proteins, have not yet been identified.

## Conclusion & future perspective

The success of *T. marneffei* in pathogenesis is due to the ability of the fungus to adapt and survive inside the host macrophage. Residing and multiplying inside the host phagosome of the host immune cells is an excellent survival strategy for this facultative intracellular pathogen to escape the adaptive immune response. Like many other facultative intracellular pathogens, *T. marneffei* uses antioxidants, heat shock proteins and well-conserved MAPK signaling cascades in response to the stresses posed by the host-cell environment. The catalase-peroxidase enzyme has been shown to be involved in the virulence of this fungus, and the deletion mutant of the gene encoding for this enzyme attenuated virulence in a macrophage infection model. However, intracellular survival mechanisms have not been well described in *T. marneffei*. Understanding the initial adaptation of the fungus to phagosomal stresses is a starting point which allows us to begin to unravel the conundrum behind. Its success will increase our understanding the pathogenesis process in this emergent pathogenic fungus.

**Executive summary**

- *Talaromyces marneffei* is an intracellular pathogenic dimorphic fungus which causes disseminated infection in human. To establish infection, the conidia must be able to adapt to conditions of heat, oxidative/nitrosative stress and nutrient deprivation found inside the phagosomes of macrophages.
- In response to oxidative and nitrosative stresses, *T. marneffei* could secrete antioxidant molecules to neutralize reactive oxygen species/reactive nitrogen species. These include superoxide dismutase, catalase-peroxidase, YapA (yeast activating protein-1) transcription factor, component of the high-osmolarity glycerol MAPK signaling pathway (encoded by *sakA* gene) and basic region leucine zipper type transcription factor (encoded by *atfA* gene). The YapA and SakA are essential in the response to oxidative and nitrosative stresses.
- Incubation at 35–37°C is the main factor to stimulate the phase transition in *T. marneffei*. Two heat shock proteins, Hsp70 and Hsp30, were characterized and are suggested to be putative contributing factors in thermal endurance. Hsp30 has been shown to accumulate inside conidia. It may be involved in conidial germination or the phase transition process, and therefore it is a putative virulence factor mediating the fungal phase conversion. In addition, SakA is required for yeast cell transition and under heat shock at 39°C.
- In response to nutrient deprivation, *T. marneffei* responds to glucose starvation during infection by upregulating genes encoding for several enzymes in gluconeogenesis and the glyoxylate cycle.
- A fuller understanding regarding the initial adaptation of *T. marneffei* to phagosomeal stresses is a starting to unravel the process of pathogenesis in this emergent pathogenic fungus.
